# The Year's Work: "The Hospital's" Review of 1913

**Published:** 1914-01-03

**Authors:** 


### THE YEAR'S WORK.

### "The Hospital's" Review of 1913.

LOOKING back on the achievements of the past year from the institutional point of view, it seems to have been marked by several great controversies and the inauguration of great changes in hospital life and work. Neither the hospitals themselves nor the great Funds which play so important a part in the voluntary system have been exempt from disputations. In the first place, it must be mentioned, the National Insurance Act came into force with the beginning of benefits, which first became payable on January 15, 1913. Then the year is noteworthy for the settlement of the dispute which had estranged St. George's Hospital from King Edward's Hospital Fund for London, until only of late the institution wisely gave way, and has returned to the system of keeping its accounts on the lines which the Fund has laid down for the purpose of comparison between the different institutions.

This controversy, settled at last after how many delays and procrastinations, has not proved the only controversy in which the great Funds have been engaged. The constitution of the Metropolitan Hospital Sunday Fund for London was also called in question, and the whole of the revised laws, which were drawn up in pursuance of the Council's resolution of January 29, were carried, and were given in THE HOSPITAL of June 28, 1913, page 398. The difficulties and mistakes which preceded this revision were duly recorded in our columns, but in a review and a summary it is more profitable to dwell on the result, which, there is reason to believe, will have proved sufficient to allay the anxieties and discontent that had previously existed among the constituents to such a degree as to endanger the very existence of Hospital Sunday. It is now time to turn our attention to the controversy which involved the Hospital Saturday Fund—namely, that induced by the claim that the hospitals which received grants from the Fund should suspend their almoner system of inquiries in favour of everyone who appeared for treatment on the recommendation of a Saturday Fund letter. We pointed out that the amount contributed by the Saturday Fund was not sufficient to admit them to a position not accorded to other contributors; and, further, if the Fund regarded its grant not as a gift, but as a payment for services to be rendered, then the hospitals would refuse it, or pointing out that every other donor was similarly protected from abuse. In September we announced that the London Hospital had refused to agree to such conditions, and in October resolutions were passed for assessing the awards in the usual way. Another dispute of interest was that now, at last, ended, over the Royal Victoria, Newcastle, Infirmary chapel. Litigation decided that while the consecration of the chapel may have been *ultra vires*, the fact of consecration devoted the building to the exclusive use of the Established Church, and the chapel has therefore now been reopened for this section of worshippers. From these subjects attention may be turned to the most important of the new buildings which have been opened or appropriated during the past year. First place must, perhaps, be given to the new King's College Hospital, opened by the King in August, and ready for work by October; to the new buildings at Guy's Hospital, which, opened by Mr. A. J. Balfour, gave new medical and dental schools to the hospital, and completed a scheme of ten years' work. Then allusion must be made to the laying of the foundation-stone last July of the South London Hospital for Women, of which the out-patient department had been opened in April. Then we may note the St. Andrew's Hospital, Dollis Hill, now taking in paying patients; the new pathological block at the Radcliffe Infirmary, Oxford; and the opening by the Queen of the new Canadian Hospital for Consumptive Children at Toronto, Canada, now known as Queen Mary's Hospital. Mention must also be made of the King's visit to the Royal West Sussex Hospital, Chichester, which has been reconstructed, and to the opening of the New Central London Ophthalmic Hospital in Judd Street; and also of the new buildings of the London School of Tropical Medicine, which we were able to announce in September as practically complete, and which owe the possibility of their existence to the success which attended Mr. Austen Chamberlain's appeal. The new out-patient department of the Royal Free Hospital began to take practical shape with the laying of the foundation-stone by Princess Christian in May.

In this connection, moreover, the removal of Westminster Hospital and its proposed amalgamation with St. George's Hospital, if a suitable arrangement to satisfy the needs of all parties over the new joint site can be arrived at, has loomed large on the horizon of London during the latter part of the year. It marks the public recognition of the hospital needs of Greater London, and the squeezing towards the periphery of the capital of those patients and the classes from which they come, which once occupied the localities in which the great central hospitals were founded. A change also must be recorded in connection with the sale of Mount Vernon Hospital at Hampstead, and the concentration on the sanatorium at Northwood, the appointments at which led to a serious dispute and the resignation of some of the medical staff. As this action on the part of the staff was accompanied by a hint that legal action might be taken against the hospital's authorities for the redress of the "summary dismissal" of which certain members complained, we are reminded of the case of Eddowes *v.* St. John's Hospital, in which the plaintiffs were awarded £50 damages for dismissal without due notice. Mr. Justice Pickford's decision, however, did not lay down the law as to whether or not a medical staff once appointed is appointed for life (save in the case of incompetence or misconduct); it merely decided that such appointments must be terminable on proper notice. It gave no colour to the contention that staff appointments, in the absence of limiting conditions, are lifelong and irrevocable

In the sphere of mental hospital activity change and movement have been equally apparent. In the first place, the passing of the Mental Deficiency Act has launched a new legislative barque which has probably as many perils to encounter as any of the members of the present Parliamentary fleet. It has led to the creation of a new Board of Control, two of the members of which contributed articles to the Special Mental Hospital Number that we published on October 11. The one certain thing about the new Act is that if any attempt to carry out its provisions on a national scale is seriously made, the expense involved will startle the country by its huge proportions. Another question in which the Government shows signs of engaging is the question of research in mental hospitals, now too well recognised as neglected, haphazard, and unco-ordinated. In this connection Mr. Reginald McKenna's visit to Cardiff Mental Hospital and his inspection of the research work there carried on is noteworthy. The need for a co-ordinating scheme has at least received official recognition. Another side of the research question is the status of the assistant medical officers in mental hospitals; for the restrictions and lack of prospects under which they suffer is partly the cause and partly the effect of an institutional life, much overwhelmed with spiritless routine and little calculated to preserve early enthusiasm. A significant result of the year has been the formation of a new Association of Assistant Medical Officers, of which Dr. F. J. Stuart, of Berrywood, Northampton, has been one of the most active promoters. The new Association hopes that combination will do for the status of assistant medical officers what it has done for attendants, and indeed for every class in and outside the hospitals which have adopted this principle. At the same time, we note the continued upward tendency of the salaries paid to attendants in mental hospitals, of which there have been several instances during the year, and the general amelioration of their lot continues to occur. It is hardly necessary to remind our readers that attendants with their organisation possess, as a class, the facilities for married life, for which the till recently unorganised assistant medical officers are now struggling. Significant, however reckless and ill-advised in this connection, was the threatened strike of mental hospital workers in the West Riding. For such a thing to be talked of—organisation, or class consciousness as the Socialists call it, must have been developed to a considerable degree.

The Poor-Law institutions have been learning that the Insurance Act has tended to increase rather than diminish the number of their patients, and perhaps even more than the hospitals have felt the growing difficulty of finding suitable resident medical officers. There has again been demonstrated the need for more frequent medical inspection, and for more numerous inspectors, so that medical superintendents may have ready official support in bringing pressure to bear upon recalcitrant Boards of Guardians. As usual, several glaring instances of overcrowding have been recorded during the year. In London there seems at last a chance that the old bitterness between the Southwark Guardians and Guy's Hospital may be healed by the action of the former in supplying an emergency station, since the King's Fund has supported the hospital's policy in refusing to overcrowd its wards and to accept chronic cases. A serious dispute arose at Camberwell over misunderstandings among the medical staff of the infirmary, and an outbreak of enteritis at the Southwark Infirmary has led to recriminations between the Southwark Guardians and the Camberwell Borough Council, since the Council owned a refuse-heap from which the Guardians were advised that the infection proceeded. The Report of the Departmental Committee on Poor-Law Orders has been issued and created much discussion, the general feeling being that improvements and defects are fairly mingled, and that nothing which falls short of the complete separation of the infirmaries from the workhouses can be satisfactory. We discussed its institutional points at length in our issues of August 23 and 30.

Two matters of great importance we have reserved to the end. The allegations made in our columns against York County Hospital by two former members of its medical staff, the public inquiry which was held by Sir Cooper Perry into the charges preferred, and the delayed report of the hospital's committee upon them, were undoubtedly events of unique importance, and showed the power of responsible journalism on the one hand, and the evils which may result from routine habits and a slack administration on the other. The exposure of certain abuses and the inquiry which followed will long make the year 1913 memorable in hospital annals. The second event was the Conference of the British Hospitals Association at Oxford, where, thanks to Sir W. Osler's lead, the whole voluntary system was overhauled and the need for provision for pay patients of small means and for adequate clinical laboratories was enforced after an active discussion. At Cardiff a medical school is now in the making, and in London attention has been fixed on the quinquennial appeal of the London Hospital, the still-delayed solution of the ambulance question, the proposals for the regulation and control of nursing homes and massage establishments, and the great bequest of the late Sir Julius Wernher of nearly half a million to the King's Fund. Death has removed Sir Edmund Hay Currie, so long secretary of the Sunday Fund; Mr. Storrs Fry, of Bristol; Mr. Guntrip King, and Mr. W. Thraves, of Chesterfield; and the autumn saw the publication of Sir E. T. Cook's masterly "Life of Florence Nightingale," and Dr. Wrench's "Lord Lister."

